# Correlation Between Pre-treatment Collateral Status and Short-Term Functional Outcome in Patients With Mild to Moderate Stroke After Reperfusion Therapy in a Local Primary Stroke Center in the Southwestern Part of Saudi Arabia

**DOI:** 10.7759/cureus.33997

**Published:** 2023-01-20

**Authors:** Saeed A Alqahtani, Ibrahim Alnaami, Adel Alhazzani, Fawziah Alahmari, Yasser Wassel, Enas Elsayed, Ahmed Abdrabou, Al-Amir Bassiouny Mohamed

**Affiliations:** 1 Neurology Section, College of Medicine, King Khalid University, Abha, SAU; 2 Neurosurgery Section, College of Medicine, King Khalid University, Abha, SAU; 3 Neurosciences Department, King Faisal Specialist Hospital and Research Center, Riyadh, SAU; 4 Neurology Section, College of Medicine, Mansoura University, Mansoura, EGY; 5 Neurology Section, College of Medicine, Zagazig University, Zagazig, EGY; 6 Radiology Department, College of Medicine, Ain Shams University, Ain Shams, EGY; 7 Neurology and Psychological Medicine Department, Faculty of Medicine, Sohag University, Sohag, EGY

**Keywords:** modified rankin scale(mrs), nihss (national institutes of health stroke scale), neurological outcome, collateral status, ais (acute ischemic stroke)

## Abstract

Background

Stroke is a substantial cause of disability and mortality worldwide and is characterized by the sudden onset of acute neurological deficit. During acute ischemia, cerebral collateral circulations are crucial in preserving blood supply to the ischemic region. Recombinant tissue plasminogen activator (r-tPA) and endovascular mechanical thrombectomy (MT) are the primary standards of care for acute recanalization therapy.

Methodology

From August 2019 through December 2021, we enrolled patients treated in our local primary stroke center with anterior circulation acute ischemic stroke (AIS) treated with intravenous thrombolysis (IVT) with or without MT. Only patients diagnosed with mild to moderate anterior ischemic stroke, as measured by the National Institutes of Health Stroke Scale (NIHSS), were included in the study. The candidate patients underwent non-contrast CT scanning (NCCT) and CT angiography (CTA) at admission. The modified Rankin scale (mRS) was used to assess the functional outcome of the stroke. The modified Tan scale, graded on a scale of 0-3, was used to determine the collateral status.

Results

This study comprised a total of 38 patients who had anterior circulation ischemic strokes. The mean age was 34. 8±13. All patients received IVT; eight patients (21.1%) underwent MT following r-tPA. In 26.3% of cases, hemorrhagic transformation (HT), both symptomatic and asymptomatic, was evident. Thirty-three participants (86.8%) had a moderate stroke, whereas five participants (13.2%) had a minor stroke. With a P-value of 0.003, a poor collateral status on the modified Tan score is substantially associated with a short, poor functional outcome.

Conclusion

In our study, patients with mild to moderate AIS with good collateral scores at admission had better short-term outcomes. Patients with poor collaterals tend to present with a disturbed level of consciousness more than patients with good collaterals.

## Introduction

The term "stroke" refers to the sudden onset of a neurological deficit caused by an acute focal injury to the central nervous system due to a vascular reason [1].

Strokes have a significant financial burden and are a primary cause of disability, dementia, and mortality worldwide. Worldwide, one in four adults will experience a stroke at some point in their lives [2]. A recent update on stroke statistics shows that stroke incidence has steadily risen in low- and middle-income countries [3]. These nations were home to 87% of all stroke-related disabilities and 70% of all stroke-related deaths [3,4].

Better outcomes and reduced mortality are associated with early revascularization to rescue salvageable cerebral tissue [5-8]. For the last decades, intravenous (IV) administration of recombinant tissue plasminogen activator (rt-PA) has been considered the primary modality of recanalization therapy in acute ischemic stroke [5,9]. Nonetheless, mechanical endovascular clot retrieval has recently been approved and has shown effectiveness in several clinical trials [6-12]. Recanalization with IV rt-PA was only successful in less than 20% of instances in a proximal middle cerebral artery (MCA), internal carotid artery (ICA), or basilar artery occlusions [13,14]. While in large-vessel occlusion cases, considerable reperfusion is achieved in 70-80% of cases by utilizing thrombectomy techniques [15].

If the main artery fails due to thrombosis or any other pathological process resulting in hemodynamic compromise, the collaterals are arterial vessels forming a subsidiary network of vascular channels to sustain blood supply to the brain tissue [16].

Primary and secondary collateral pathways are formed when extracranial and intracranial arteries converge. The Circle of Willis supplies the primary collaterals with arteries while the ophthalmic artery and leptomeningeal vessels contribute to the formation of the secondary collaterals [16]. The Circle of Willis, which makes up most of the brain's collateral network, can maintain perfusion as soon as a major artery is blocked. However, when occlusion occurs in a distal intracranial artery, the Circle of Willis cannot compensate for the CBF reduction. The secondary flow through leptomeningeal anastomoses is the principal collateral [15,17].

Blood can flow in both directions in leptomeningeal collaterals, which allows for retrograde perfusion of nearby regions and the preservation of a functional area of brain tissue known as the "ischemic penumbra." Blood flow is sufficiently decreased in the ischemic penumbra to stop physiological function but not to the point where it results in irreversible cellular death [17]. During acute ischemia, the formation of cerebral collateral circulations is crucial in preserving blood supply to the ischemic region. Of note, the degree of infarction size, the response to recanalization, the likelihood of hemorrhagic transformation after reperfusion, and the overall neurological outcome have all been influenced by the collateral status [18,19]. To assess the condition and grade the collaterals, a variety of neuroimaging modalities are used, such as single- or multi-phase CT angiography [20-23], CT or MR perfusion imaging [24-26], fluid-attenuated inversion recovery imaging [27,28], and digital subtraction angiography, which is the gold standard but has limited application [29-30].

We conducted this study to investigate the correlation between collateral status and functional outcome in patients with mild to moderate anterior circulation stroke treated with IVT with or without MT.

## Materials and methods

Patients with anterior circulation ischemic stroke treated with IVT with or without MT were enrolled in this follow-up study from August 2019 through December 2021. Patients with AIS admitted to the Neurology department at King Fahad Armed Forces hospital in the southern region of Saudi Arabia and older than 18 years, as well as those who had received IVT with rt-PA with or without MT, all met the inclusion criteria. Only people with an ischemic stroke that the NIHSS classified as mild to moderate were included.

Patients with walking-up stroke were excluded from the study. Patients with disorders including concomitant carotid stenosis or dissection, aortic dissection, hypotension related to cardiac reasons, drug side effects, or infections that compromise collateral blood flow were excluded from this study. Transient ischemic attack patients were also disqualified from this study. According to food and drug administration (FDA) recommendations, all included patients received an identical 0.9 mg/kg intravenous tPA dosage.

In our study, we used the modified Rankin scale (mRS) to assess the functional outcome of our stroke patients three months after the event. This scale goes from 0 to 6, with 0 representing no symptoms and 6 representing deaths. Thus, patients who scored 0 to 2 were classified as having a favorable (good) outcome while those who scored 3-6 had an unfavorable (bad) outcome [7].

The NIHSS assessed the stroke severity upon admission and classified it as no symptoms, 0; minor stroke, 1-4; moderate stroke, 5-15; moderate to severe stroke, 16-20; and severe stroke, 21-42. A drop or increase of 4 points from baseline on the NIHSS at 24 hours was considered an early sign of clinical improvement or deterioration [8]. Following a thorough diagnostic workup, stroke subtypes were identified using the Trial of Org 10172 in Acute Stroke Treatment (TOAST) criteria [26]. Cases with carotid artery stenosis were disregarded. Early CT changes were defined according to European Cooperative Acute Stroke Study (ECASS II) [27]. A 24- to 36-hour control CT was performed to identify the hemorrhagic transformations based on the ECASS II criteria [27]. The ABC/2 rule calculated the infarct volume in the control CT scan [28].

Neuroimaging protocol

All patients will get a non-contrast CT scan (NCCT) at the time of admission to rule out bleeding (Definition AS, Siemens, Erlangen, Germany). The weight-adjusted bolus is given right after NCCT if the patient qualifies for an intravenous tissue plasminogen activator (tPA). Early ischemia alteration in the brain was detected ASPECTS (using the Alberta stroke program early CT score) system [29]. Acquisitions of NCCT and CTA were carried out in accordance with established departmental procedures. CTA was performed after a 25-second delay and administration of 80 mL of a contrast agent (Omnipaque350) at an injection rate of 4 mL/s by using a power injector. Images were obtained from the C3 vertebral body level through the Circle of Willis. Afterward, source images were reconstructed into standardized maximum-intensity projection views of the intracranial vasculature. A radiologist with extensive experience evaluated all CTA images. Collateral status CTA source images were assessed using the modified Tan scale, which yielded scores between 0 and 3. A score of 0 meant that there was no collateral supply to the MCA territory that was blocked. A score of one indicated collateral supply filling ≤50% but >0% of the occluded MCA territory. A score of two was given for collateral supply filling >50% but <100% of the occluded MCA territory. A score of three was given for 100% collateral supply of the occluded MCA territory [30]. Collateral status was classified as “good” if seen in ⩾50% of the MCA territory and “poor” if seen <50% of the territory [30].

Statistical analyses

Statistical Package for the Social Sciences was used to conduct the statistical analysis (version 28.0; IBM Corp, Armonk, NY). For the quantitative variables, the findings were presented as mean standard deviation (SD), and for the categorical variables, they were presented as percentages. For continuous data, the student t-test was employed, and for categorical variables, the chi-square test. When more than 20% of cells have predicted frequencies below 5, Fisher's exact test was applied. To investigate several factors influencing the functional outcome in our series, multivariate logistic regression analysis was utilized. The threshold for statistical significance was set at P <0.05.

## Results

The study comprised a total of 38 patients with anterior circulation ischemic stroke. The mean age was 34.8±13. Only eight patients (21.1%) underwent MT after rt-PA, and all patients received IVT. About twenty-six percent (26.3%) of patients had a hemorrhagic transformation, both symptomatic and asymptomatic. The most frequent form of stroke (on NIHSS) was moderate (78.9 percent), followed by minor (5.3%) and moderate to severe (15.8%) strokes (Table 1).

**Table 1:** Admission Baseline Characteristics of the Participants ASPECT = The Alberta stroke program early CT score; mRS = modified Rankin Scale; CT = Computed Tomography; NCCT = Non-Contrasted CT; NIHSS = National Institutes of Health Stroke Scale; PCI = Percutaneous Coronary Intervention; MCA = Middle Cerebral Artery; ACA = Anterior Cerebral Artery; TOAST = Trial of Org 10172 in Acute Stroke Treatment; SD = Standard Deviation

Variables	Number	Percent
Age (years, mean ± SD)		34.8±13
Gender		
Male	29	76.3
Female	9	23.7
Clinical		
Right hemiplegia	25	65.8
Left hemiplegia	13	34.2
Smoking	9	23.7
Hypertension	27	71.1
Diabetes mellitus	15	39.5
Cardiac disease	17	44.7
Atrial fibrillation (AF)	7	18.4
PCI	5	13.2
TOAST		
cardio embolism	9	23.7
Small artery stroke	29	76.3
Stroke severity on NIHSS		
Minor Stroke	5	13.2
Moderate	33	86.8
CT findings		
Normal	12	31.5
Early ischemic changes	7	18.4
Recent infarction	14	36.8
Old infarct(s)	5	13.2
CT ASPECT on NCCT		
<=7	8	21.1
>=8	30	78.9
Edema mass effect	1	2.6
Hemorrhagic transformation	10	26.3
Modified Tan score		
Poor collateral	7	18.4
Good collateral	31	81.6
CT angiography (CTA)		
Patent vessels	20	52.6
MCA or its branch	12	31.6
ACA or its branch	6	15.8
Mechanical thrombectomy	8	21.1
Craniectomy	3	7.9
Functional outcome (by mRS)		
Favorable	14	36.8
Unfavorable	24	63.2

Good collaterals were present in 81.6% percent of the cases where the collateral status was evaluated using the modified Tan score. In measurements of the short-term result of stroke, a good outcome (mRS score 0-2) was present in 36.8% of cases, whereas a negative outcome was found in 63.2%. There was a statistically significant association between unfavorable outcomes and conscious level, stroke severity by NIHSS, and the presence of ischemic heart disease (Table 2).

**Table 2:** Demographic, Clinical, and Radiological Characteristics of Stroke Patients With Favorable and Unfavorable Outcomes AF = Atrial Fibrillation; ASPECT = The Alberta stroke program early CT score; CABG = Coronary Artery Bypass Graft; CT = Computed Tomography; CTA = CT Angiography; NCCT = Non-Contrasted CT; NIHSS = National Institutes of Health Stroke Scale; PCI = Percutaneous Coronary Intervention; SBP = Systolic Blood Pressure; DSP = Diastolic Blood Pressure; MCA = Middle Cerebral Artery; ACA = Anterior Cerebral Artery; TOAST = Trial of Org 10172 in Acute Stroke Treatment; SD = Standard Deviation

	Favorable N=14	Unfavorable N=24	P-Value
Age (mean ±SD)	57.4±11.1	67.1±13.4	0.027
Sex			0.067
Male	13(92.9%)	16 (66.7%)	
Female	1 (7.1%)	8 (33.3%)	
Clinical features			0.5
Right Hemiplegia	10 (71.4%)	15 (62.5%)	
Left hemiplegia	4 (28.6%)	9 (37.5%)	
Onset-treatment time, min (Door to needle time) mean ±SD	55.36±7.459	58.75±8.999	0.24
Consciousness			
Intact	11 (78.6%)	9 (37.5%)	
Disturbed	3 (21.4%)	15 (62.5%)	0.014
TOAST			
Cardioembolic stroke	1 (7.1%)	8 (33.3%)	0.6
Small artery stroke	13 (92.9%)	16 (66.7%)	
NIHSS			
Minor to mild	4 (28.6%)	1 (4.2%)	
Moderate	10 (71.4)	23 (95.8%)	0.032
NIHSS (mean ± SD)	7.29± 2.6	11.92± 2	<0.001
Smoking	8 (57.1%)	19 (79.2%)	0.8
Hypertension	8 (57.1%)	19 (79.2%)	0.149
Admission SBP, mm Hg (Mean ±SD)	147.6±32	162.6±24.8	0.16
Admission DBP, mm Hg (Mean ±SD)	DBP82.25±11.725	85±14.5	0.2
Diabetes Mellitus	6 (42.9%)	9 (37.5%)	0.74
Cardiac disease			
Non-cardiac	10 (71.4%)	11 (45.8%)	
Ischemic heart disease	2 (14.3%)	10 (41.7%)	0.040
CABG	2 (14.3%)	0 (0.0%)	
AF	0 (0.0%)	3 (12.5%)	
PCI	0 (0.0%)	5 (20.8%)	0.067
Carotid Stenosis	0 (0.0%)	4 (16.7%)	0.106
CT findings			
Normal	9 (64.3%)	3 (12.5%)	
Early ischemic changes	0 (0.0%)	7 (29.2%)	0.002
Recent Infarction	5 (35.7%)	9 (37.5%)	
Old Infarction	0 (0.0%)	5 (20.8%)	
CT ASPECT on NCCT			
<=7	0 (0.0%)	8 (33.3%)	0.01
>=8	14 (100.0%)	16 (66.7%)	
Infarction volume			
CTA			
Patent	12 (85.7%)	8 (33.3%)	
MCA or it's branch	0 (0.0%)	12 (50.0%)	
ACA or it's branch	2 (14.3%%)	4 (16.7%%)	0.002
Collateral status by Modified Tan score			
Good collateral	14 (100.0%)	13 (54.2%	
Poor collateral	0 (0.0%)	11 (45.8%)	0.003

CT ASPECT <=7 and poor collateral status on modified Tan score are significantly related to unfavorable outcomes with P-values 0.01 and 0.003, respectively. Poor collateral status is a predictor of short-term stroke outcome on univariate logistic regression, which is not the case after controlling for age, initial NIHSS, and conscious level, indicating that poor collateral status is a dependent variable (Table 2).

## Discussion

Stroke is a major health issue worldwide [1]. Persistent anterior cerebral circulation occlusion is one of the most devastating clinical events, often causing severe neurologic deficits or death [2]. Previous studies reported that assessment of baseline or pre-treatment collateral status has potential value in patient selection and prognosis of AIS patients receiving IVT and/or endovascular thrombectomy (EVT) [29].

The male predominance in our study has been reported and demonstrated in earlier studies in Saudi and still exists, where almost three-quarters of the study population are males [31]. The fact that 43% of the study population is diabetic and one-quarter of them hypertensive represents the actual incidence of both diseases in our Saudi population, where both are well-known as significant risk factors for strokes; however, smoking is reported by almost one-quarter of the study patients. Small artery strokes are more noticeable in the Saudi population, which is compatible with earlier studies on the Saudi people, which explains that a small percentage of patients underwent mechanical thrombectomy [31].

The ASPECT score was eight or more in 30 patients, which was the independent variable in the presence of good collaterals and even the prediction of outcome. In contrast, the good ASPECT score did not stand in the prediction of favorable versus unfavorable outcomes, where almost 50% of the patients with good collaterals ended up with favorable outcomes despite the good ASPECT score at presentation.

Good collateral circulation has a considerable impact on patients' prognoses and favorable neurological outcomes [29]. The compensating collateral flow is essential for maintaining blood flow to the benign oligemia and penumbra and preventing the progression of brain ischemia in AIS with arterial occlusion. Patients with stronger collateral circulation may experience fewer severe neurological symptoms at baseline and achieve better functional results [30].

Studies with a longer time window, such as those with a 4.5-7-hour time window, tended to show more substantial protective benefits of good collaterals on the functional outcome. The fact that patients with poor baseline collaterals, who have little salvageable tissue to be reperfused beyond 4.5 hours, have a relatively low rate of good functional outcomes.

Previous investigations have used penumbra imaging to identify AIS patients who may benefit from receiving IVT treatment three to six hours after ictus. These studies, however, did not successfully extend the time window for IVT, in part because penumbra delineation was inaccurate [29]. After mechanical revascularization or rt-PA thrombolysis, patients with good collateral circulation are less likely to experience hemorrhagic sequelae, whereas those with poor collateral circulation are more likely to get HT (88.9% vs. 38.1%) [30]. Patients with good collaterals experienced higher rates of recanalization following IVT with rt-PA than those with poor collaterals (61.8% vs. 28.1%) [30]. For a better understanding of the influence of the pre-treatment collateral score on the long-term functional result of AIS, additional long-term follow-up studies are needed.

## Conclusions

Good collateral circulation at the baseline substantially influences patients' prognoses and favorable neurological outcomes during acute cerebral ischemia. Patients with mild to moderate acute ischemic stroke with good collateral scores who received reperfusion therapy at admission had better short-term outcomes. In our study, patients with poor collaterals tend to present with a disturbing level of consciousness more than patients with good collaterals. Care for selecting patients with poor collaterals for mechanical thrombectomy is advised.
